# Dynamic Expression of the Type 1 Brugada ECG Pattern During Tilt Table Testing Using Continuous High Precordial Lead Positioning

**DOI:** 10.3390/diagnostics16111718

**Published:** 2026-06-03

**Authors:** Eduardo Nolla Silva Pereira, Luciana Sacilotto, Tan Chen Wu, Gabrielle D’Arezzo Pessente, Nemer Luis Pichara, Denise Tessariol Hachul, Mauricio Ibrahim Scanavacca, Francisco Carlos da Costa Darrieux

**Affiliations:** Cardiac Arrhythmias Unit, Instituto do Coração—InCor-HCFMUSP, Faculdade de Medicina, Hospital das Clínicas da Universidade de São Paulo, São Paulo 05403-900, Brazil; lu.sacilotto@gmail.com (L.S.); tanchencardio@gmail.com (T.C.W.); gabrielle.pessente@hc.fm.usp.br (G.D.P.); nlpichara@gmail.com (N.L.P.); denise.hachul@gmail.com (D.T.H.); mauricio.scanavacca@gmail.com (M.I.S.); francisco.darrieux@incor.usp.br (F.C.d.C.D.)

**Keywords:** Brugada syndrome, Brugada type-1 ECG pattern, tilt table test, high precordial leads, autonomic modulation

## Abstract

**Background/Objectives:** The Type 1 Brugada ECG pattern (BrP1) fluctuates according to autonomic influences. Tilt table testing induces changes in both sympathetic and parasympathetic activity. It may provide insights into the dynamic behavior of BrP1 when combined with high-precordial lead placement. However, the clinical significance of BrP1 variability during tilt table testing remains poorly defined. **Methods:** This cross-sectional study evaluated patients with confirmed Brugada syndrome who underwent tilt table testing with continuous ECG monitoring using high-precordial leads. BrP1 behavior was assessed during predefined phases: baseline supine position, orthostatic tilt, nitrate administration, recovery, and syncope when present. Subsequently, clinical characteristics and test results were analyzed for associations with dynamic BrP1 expression. **Results:** Forty-four patients (mean age 49 years; 72.7% men) were included. Thirty-five patients (79.5%) had a spontaneous type 1 Brugada ECG pattern, and nine (20.5%) had a drug-induced pattern. BrP1 expression varied dynamically/heterogeneously during tilt table testing. No patient without BrP1 at admission developed the pattern during tilt phases. Thirty patients (68.1%) remained negative throughout testing, while five lost the pattern during the test (11.3%), and nine (20.4%) showed persistent BrP1 in all phases. Persistent BrP1 was associated with more frequent presumed arrhythmic syncope and implantable cardioverter defibrillator implantation (*p* < 0.05). No atrial or ventricular arrhythmias or device-related complications occurred. **Conclusions:** Tilt table testing with high precordial leads does not unmask BrP1 and should not be used as a diagnostic provocation tool. However, it allows for the characterization of autonomic modulation and phenotypic stability in Brugada syndrome. Any potential prognostic relevance of dynamic BrP1 behavior remains speculative and requires evaluation in adequately powered prospective studies.

## 1. Introduction

Brugada syndrome (BrS) is an autosomal dominant inherited disorder characterized by coved-type ST-segment elevation in the right precordial leads and an increased risk of ventricular arrhythmias [1,2]. Both arrhythmic events and the type 1 Brugada pattern (BrP1) are known to occur preferentially under conditions of heightened vagal tone [3,4]. High right-precordial lead placement is also an established technique to enhance detection of BrP1 [5,6]. Tilt table testing can induce marked vagal stimulation, particularly during the recovery phase and following syncope [7,8]. Nevertheless, the characteristics of the electrocardiographic phenotype during tilt table testing with high precordial lead placement in patients with Brugada syndrome are not yet well defined.

## 2. Materials and Methods

### 2.1. Study Design

This is a cross-sectional study with data analysis aimed at evaluating electrocardiographic pattern in high right-precordial leads of patients with BrS throughout all phases of the tilt table test and determining whether the test can unmask BrP1.

The study was approved by the Institutional Ethics Committee of Hospital das Clinicas da Universidade de São Paulo (protocol number 54223421.8.0000.0068), ensuring compliance with the ethical principles outlined in the Declaration of Helsinki.

Inclusion criteria: All patients were ≥18 years old and had a confirmed diagnosis of BrS, documented by at least one ECG demonstrating a BrP1, either spontaneously or drug-induced. BrP1 was defined as a J-point elevation ≥2 mm in high right or left precordial leads (2nd, 3rd, or 4th intercostal spaces), accompanied by a coved-type ST-segment elevation and negative T waves. Written informed consent was obtained from all participants.

Exclusion criteria: Prior BrS ablation and ventricular arrhythmic events within the previous 6 months.

Clinical data were obtained from all patients, including demographic and relevant medical history information. Genetic testing, ajmaline provocation, and electrophysiological study (EPS) when available were also reviewed.

### 2.2. Tilt Test Protocol

The test was conducted in a quiet, dimly lit room. Patients underwent continuous noninvasive blood pressure monitoring and continuous electrocardiographic recording using electrodes placed in the 2nd, 3rd, and 4th right and left intercostal spaces. When nitrate was administered, a dose of 1.25 mg of sublingual isosorbide dinitrate was used.

**Figure 1:**
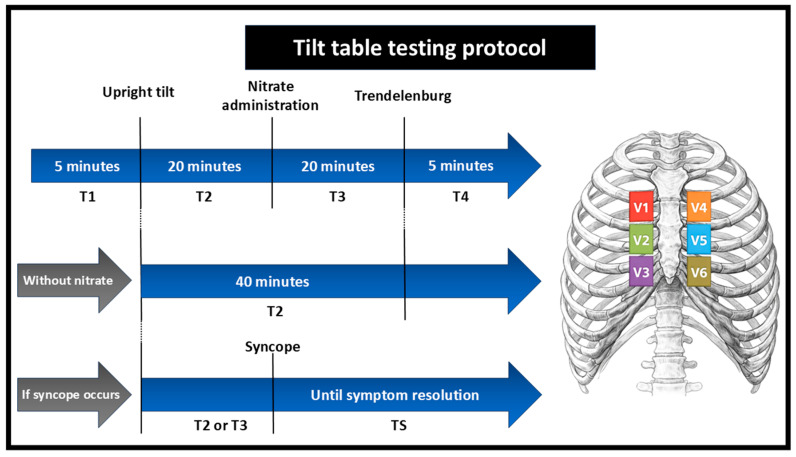
Tilt table testing protocol. Schematic representation of the tilt table test phases, including baseline supine position (T1), upright tilt (T2), nitrate administration when used (T3), and recovery in Trendelenburg position (T4). Alternative pathways are shown for protocols performed without nitrate and for tests interrupted due to syncope. High precordial leads were utilized throughout the protocol. T1: resting supine baseline. T2: first 20 min of orthostatic tilt or 40 min if no nitrate was administered. T3: 20 min after sublingual nitrate administration. T4: immediately after returning the table to the horizontal position in the Trendelenburg posture. TS: occurrence of vasovagal reflex and/or syncope.

During the tilt table test, electrocardiographic evaluation followed the predefined phases below (Figure 1).

Electrocardiographic changes were considered positive when a J-point elevation ≥2 mm appeared in any high precordial lead (2nd, 3rd, or 4th intercostal spaces, right or left), accompanied by coved-type ST-segment elevation with negative T waves. Disappearance of a pre-existing BrP1 at any phase of the test was also recorded. All ECGs were independently reviewed by two electrophysiologists without blinding. In cases of disagreement, a third electrophysiologist provided blinded adjudication.

### 2.3. Statistical Analysis

Analyses were performed using IBM SPSS for Windows, version 22.0. Qualitative variables were summarized as absolute and relative frequencies, whereas quantitative variables were described using standard summary measures (mean, standard deviation, median, and quartiles). Patients were categorized according to the electrocardiographic behavior of the BrP1 throughout the tilt test, and clinical, demographic, and disease-related characteristics were compared across groups. Associations between categorical variables and BrP1 behavior were evaluated using likelihood-ratio tests, except for age, which was compared using analysis of variance (ANOVA), following the recommendations of Kirkwood and Sterne. All analyses adopted a two-tailed alpha level of 0.05. Data tabulation was performed using Microsoft Excel 2013.

## 3. Results

### 3.1. Clinical Characteristics

A total of 44 patients underwent tilt table testing between May 2022 and September 2025. Baseline clinical characteristics and medical history are summarized in Table 1. The mean age was 49.1 years, and 32 patients (72.7%) were male. A positive family history of sudden cardiac death was observed in 29 patients (65.9%).

A spontaneous BrS pattern was identified in 35 patients (79.5%), whereas nine patients (20.5%) exhibited a drug-induced pattern. Presumed arrhythmic syncope was documented in three patients (6.8%), while non-arrhythmic syncope occurred in seven patients (15.9%). Two patients (4.5%) had a history of aborted sudden cardiac death, and nocturnal agonal respiration was reported in nine patients (20.5%).

Regarding device therapy, 17 patients (38.6%) had an implantable cardioverter defibrillator (ICD), implanted for primary prevention in 13 patients (29.5%) and for secondary prevention in four patients (9.1%). Among ICD recipients, eight patients (47.1%) experienced at least one shock. Three shocks were appropriate, whereas six were inappropriate (one patient experienced both). Causes of inappropriate shocks included T wave oversensing, atrial fibrillation, supraventricular tachycardia (*n* = 2), and lead dysfunction (*n* = 2).

### 3.2. Ajmaline Test, Genetic Testing, and Electrophysiological Study

An ajmaline challenge was performed in 14 patients, confirming the diagnosis in nine individuals with a drug-induced Brugada pattern. In five patients, although the ajmaline test was positive, the ECG pattern was later reclassified as spontaneous BrP1 after subsequent documentation of spontaneous ST-segment elevation.

Genetic testing was conducted in 30 patients (68.1%). Pathogenic or likely pathogenic variants were identified in nine patients (30%), while variants of uncertain significance or negative results were observed in 21 patients (70%).

An electrophysiological study was performed in 25 patients (56.8%). Ventricular refractory period assessment was available in 17 studies, with a refractory period <200 ms in four patients (23.5%) and ≥200 ms in 13 patients (76.5%). Inducible ventricular arrhythmias were identified in 11 patients (44%), while 14 patients (56%) did not exhibit inducible arrhythmias.

### 3.3. Dynamic Electrocardiographic Behavior During Tilt Table Testing

**Figure 2:**
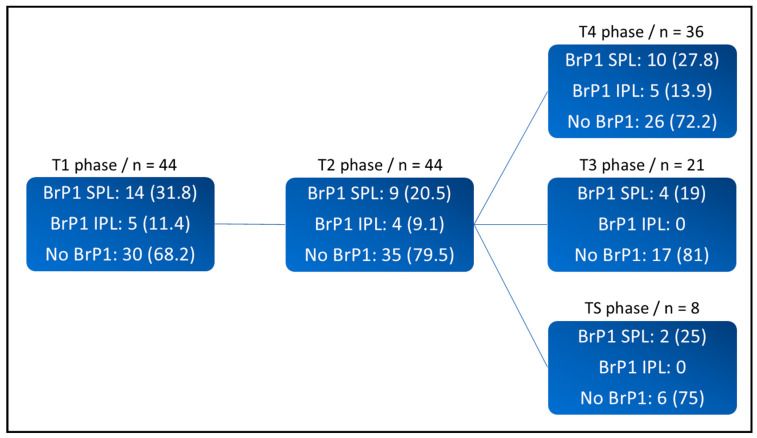
Dynamic behavior of the type 1 Brugada ECG pattern during tilt table testing. Distribution of BrP1 expression across tilt table test phases. SPL = superior precordial leads; IPL = inferior precordial leads; BrP1 = type 1 Brugada pattern; *n* = number of patients. T1 = resting supine baseline; T2 = first 20 min of upright tilt or 40 min when nitrate was not administered; T3 = upright tilt after nitrate administration; T4 = recovery in Trendelenburg position; TS = syncope phase.

Dynamic electrocardiographic changes during tilt table testing were analyzed. At baseline (T1), a BrP1 was present in superior precordial leads (SPL) in 14 patients (31.8%), of whom five also exhibited a BrP1 in inferior precordial leads (IPL). No patient demonstrated a BrP1 exclusively in IPL (Figure 2).

During the upright phase before nitrate administration (T2), a BrP1 was observed in SPL in nine patients (20.5%), with concomitant involvement of IPL in four of these patients. Disappearance of the BrP1 during T2 occurred in five patients (35.7%) in SPL and in one patient (20%) in IPL.

The nitrate protocol was performed in 21 tilt table tests (47.7%). Initially, nitrates were omitted from the protocol. Due to the low diagnostic yield for unmasking the type 1 Brugada ECG pattern, nitrates were later incorporated into the protocol. Among patients who underwent tilt table testing with nitrate administration (*n* = 21), four exhibited BrP1 in SPL and one in IPL during phase T2. During phase T3 (electrocardiographic assessment in the upright position after nitrate administration), BrP1 persisted in superior leads in all four patients, whereas the pattern disappeared in inferior leads in the patient who had previously exhibited IPL involvement. The remaining three patients with IPL involvement during T2 belonged to the group that did not receive nitrate.

During tilt table testing, syncope occurred in eight patients (20.5%). Among them, three patients exhibited a BrP1 pattern in SPL, and none in IPL during the T1 phase. During the upright phase, only one patient maintained the BrP1. After syncope and repositioning to the Trendelenburg position, this patient continued to exhibit the BrP1, whereas another patient demonstrated reappearance of the pattern. Of the eight syncopal events, one was classified as mixed, two as cardioinhibitory, four as vasodepressor and one as an exaggerated response to nitrate administration.

During phase T4, corresponding to the Trendelenburg position in the absence of syncope (*n* = 36; 81.2%), 10 patients (27.8%) maintained or exhibited reappearance of a BrP1 in SPL. Among these patients, five also demonstrated the pattern in IPL.

Based on changes in the BrP1 during tilt table testing, patients were classified into three groups (Table 2).

Group A comprised patients without a BrP1 at admission who remained negative throughout the test (*n* = 30; 68.2%). Although no BrP1 was present on the admission ECG, all patients in this group had a previously confirmed diagnosis of Brugada syndrome based on prior spontaneous or drug-induced documentation of a type 1 Brugada pattern.

Group B included patients with a BrP1 at admission who exhibited transient disappearance of the pattern during tilt table testing (*n* = 5; 11.4%).

Group C consisted of patients with a BrP1 at admission that persisted throughout the entire test (*n* = 9; 20.4%).

No patient was admitted without a BrP1 pattern that was subsequently unmasked during tilt table testing.

**Figure 3:**
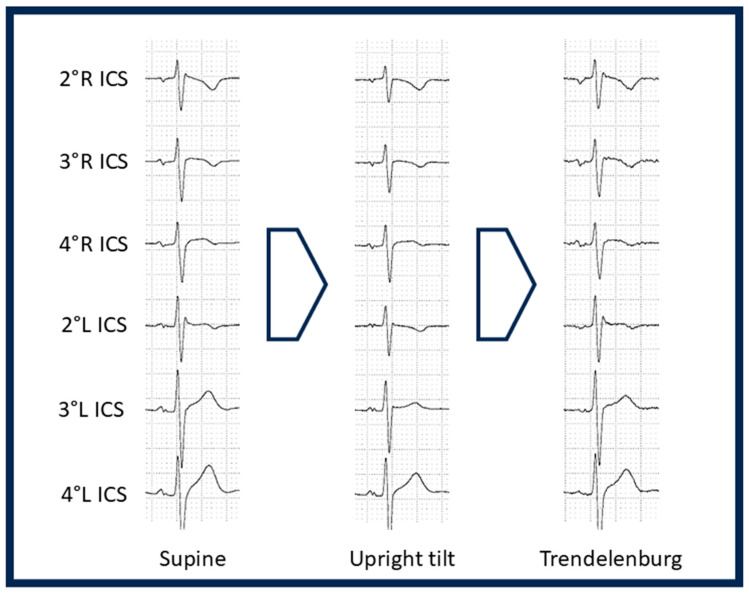
Representative patient from Group A. Despite a diagnosis of Brugada syndrome, the type 1 Brugada ECG pattern was not observed during any phase of the tilt table test. ICS = intercostal space; L = left; R = right.

**Figure 4:**
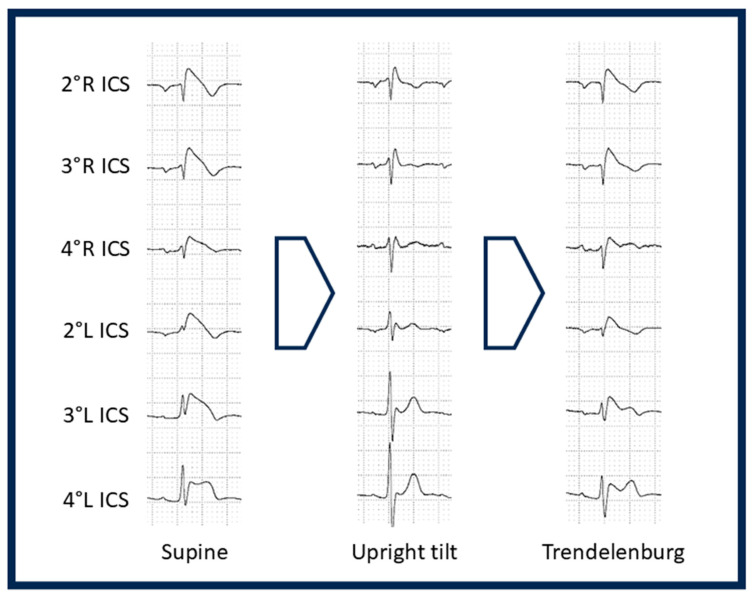
Representative patient from Group B. The patient exhibits a type 1 Brugada ECG pattern in five leads (2nd, 3rd, and 4th right intercostal spaces and 2nd and 3rd left intercostal spaces). During the upright tilt phase, the Brugada pattern disappeared in all leads and reappeared in the same leads during the recovery (Trendelenburg) phase. ICS = intercostal space; L = left; R = right.

**Figure 5:**
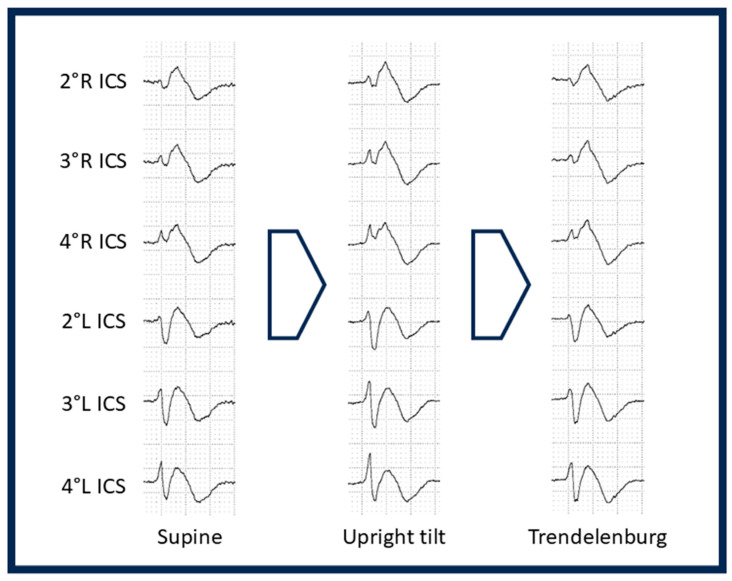
Representative patient from Group C. The patient exhibited a type 1 Brugada ECG pattern in all six leads, which persisted throughout all phases of the tilt table test. ICS = intercostal space; L = left; R = right.

Representative ECG examples of Groups A, B, and C are shown in Figure 3, Figure 4, and Figure 5, respectively. In Figure 3, the patient did not exhibit a BrP1 pattern at any time during tilt table testing. In Figure 4, the patient exhibited a BrP1 in five leads (right 2nd, 3rd, and 4th intercostal spaces; left 2nd and 3rd intercostal spaces), which disappeared during the upright phase and reappeared during recovery in the same leads. In Figure 5, the patient exhibited a BrP1 in all six leads, which persisted throughout all phases of the tilt table test.

Clinical characteristics and ancillary test results were correlated with dynamic changes in the BrP1 pattern during tilt table testing. As shown in Table 2, the presence of presumed arrhythmic syncope was significantly associated with ECG pattern variability: no arrhythmic syncope occurred in Group A, whereas its prevalence was 20% in Group B and 22% in Group C (*p* = 0.025). Implantable cardioverter defibrillator (ICD) implantation was less frequent in Groups A and B compared with Group C (26.7%, 20%, and 88.9%, respectively; *p* = 0.010).

### 3.4. Complications

No complications occurred during the 44 tilt table tests. Syncope episodes were not associated with trauma. No atrial or ventricular arrhythmias were observed. Among patients with ICD, no device-related complications or ICD shocks occurred during testing.

## 4. Discussion

This study provides the first systematic evaluation of the dynamic electrocardiographic behavior of the type 1 Brugada pattern (BrP1) during tilt table testing using high precordial leads in a cohort of patients with BrS. Previous observations addressing this topic were limited to very small samples, including an initial exploratory report from our group involving only three patients [9]. By substantially expanding these preliminary data, the present study further supports the interaction between autonomic modulation and phenotypic expression of BrS.

BrP1 expression during tilt table testing was highly dynamic, with patients demonstrating variable electrocardiographic responses across different test phases. The predominance of BrP1 expression in superior precordial leads reinforces the established diagnostic value of high precordial lead positioning in BrS and suggests that conventional 12-lead ECG monitoring alone would likely underestimate the dynamic extent of phenotypic expression during autonomic provocation [10].

Tilt table testing failed to reveal BrP1 in any patient who did not display the pattern upon initial admission. Given that the test was unable to unmask BrP1 even in patients with a confirmed diagnosis of BrS, the absence of a control group of individuals without BrS does not represent a meaningful limitation in this context, as tilt table testing would be virtually unable to induce BrP1 in patients who do not carry the underlying substrate. Therefore, the main finding of this study is that tilt table testing should not be used or recommended as a diagnostic provocation tool to unmask BrS. This is of particular clinical relevance, since patients with suspected BrS—whether due to a family history of the syndrome or presenting symptoms such as syncope or aborted sudden cardiac death—require a thorough diagnostic workup that includes serial ECG recordings with high precordial lead positioning at different time points, and, when necessary, pharmacological provocation with sodium-channel blockers such as ajmaline [1,2]. Furthermore, modified treadmill exercise testing with high precordial lead positioning has also emerged as a potential strategy for unmasking the Brugada ECG pattern [11,12].

Nitrate administration was introduced midway through the study, resulting in protocol heterogeneity across participants. The primary finding of this study—that tilt table testing failed to unmask BrP1—was consistent regardless of whether nitrates were administered. Nevertheless, the change in protocol may have introduced variability in secondary observations, particularly those related to dynamic ECG behavior during individual test phases. Given the limited sample size, stratified analyses according to nitrate use were not performed.

Dynamic changes in BrP1 expression during tilt table testing likely reflect shifts in autonomic balance [13,14]. BrS is strongly influenced by parasympathetic activity, with both ST-segment elevation and malignant ventricular arrhythmias occurring under conditions of heightened vagal tone [3,4]. In this context, the disappearance of BrP1 during orthostatic stress and its reappearance during recovery or Trendelenburg positioning support the hypothesis that sympathetic activation may attenuate phenotypic expression, whereas vagal predominance may enhance it.

An additional observation was the association between persistent BrP1 expression during tilt table testing and baseline clinical features. Patients who maintained BrP1 across all phases of the test exhibited a higher prevalence of presumed arrhythmic syncope and more frequent ICD implantation than those in whom the pattern was absent or transient. However, these findings should be interpreted with considerable caution. The subgroups analyzed were small, with some comprising as few as five to nine patients, and the number of events in certain categories—particularly presumed arrhythmic syncope—was extremely low in absolute terms. Moreover, multiple comparisons across small subgroups further increase the probability of false-positive findings. Under these conditions, even statistically significant results carry a substantial risk of type I error. In addition, ICD implantation primarily reflects prior clinical decision-making and baseline risk assessment rather than a direct arrhythmic endpoint.

Within this context, our findings may be consistent with the concept of a “Brugada burden,” whereby the extent and stability of ECG phenotypic expression may carry prognostic implications [15,16,17]. Previous studies have demonstrated that spontaneous and persistent expression of the BrP1 is associated with a worse prognosis compared with intermittent or drug-induced patterns [18]. Our findings are also consistent with the observations of Veltmann et al. [19], who prospectively demonstrated marked spontaneous fluctuation between diagnostic and non-diagnostic ECGs in patients with BrS, with only 2% of patients exhibiting a continuously diagnostic BrP1 during follow-up. Similarly, in the present study, most patients with previously confirmed BrS did not exhibit BrP1 at the time of tilt test admission, further underscoring the highly dynamic nature of phenotypic expression of this condition. It is worth noting, however, that their study did not employ high precordial lead positioning, which may have contributed to an underestimation of BrP1 prevalence in their cohort. Notably, Veltmann et al. also reported that patients with diagnostic type 1 ECGs in more than 50% of serial recordings had significantly higher rates of inducible ventricular arrhythmias during electrophysiological studies [19].

This study has some limitations. First, only 44 patients from a single center were included, which may restrict the generalizability of our findings. Furthermore, as previously discussed, the resulting subgroup sizes were small, with very few events in certain categories, which limits the robustness of subgroup comparisons. Second, the cross-sectional design did not allow for assessment of long-term outcomes or evaluation of the prognostic implications of dynamic BrP1 behavior during tilt table testing. Third, nitrate administration was introduced partway through the study protocol because of the low initial diagnostic yield, resulting in heterogeneous test conditions across participants. However, this reflects the exploratory and hypothesis-generating nature of the study and the iterative refinement of the protocol during its course. Fourth, not all participants underwent genetic testing, ajmaline provocation, or electrophysiological studies, thus limiting the comprehensive assessment of associations between clinical or genetic factors and dynamic BrP1 behavior. Fifth, the initial ECG interpretation was performed without blinding to the procedural context, which may have introduced observer bias in the assessment of dynamic BrP1 behavior, although independent review and blinded adjudication in cases of disagreement were performed to mitigate this limitation. Future investigations should address these limitations by adopting larger prospective multicenter studies, with standardized protocols, comprehensive ancillary testing, and extended follow-up, to clarify the clinical and prognostic significance of dynamic Brugada ECG behavior during tilt table testing.

Due to the limited sample size and small subgroup distribution, multivariate analysis was not feasible without a substantial risk of model overfitting and unreliable estimates. Accordingly, all subgroup comparisons are based on univariate analyses.

## 5. Conclusions

Tilt table testing with high precordial leads does not unmask BrP1 and should not be used as a diagnostic provocation tool. However, the test corroborates previous evidence that autonomic modulation influences the electrocardiographic pattern in Brugada syndrome. Persistent BrP1 expression throughout tilt table testing was associated with presumed arrhythmic syncope and more frequent ICD implantation.

## Figures and Tables

**Table 1:** Baseline characteristics of the patients.

Variable	Descriptive (*n* = 44)
Age, years, mean ± SD	49.1 ± 14.7
Male sex, *n* (%)	32 (72.7)
Spontaneous BrS, *n* (%)	35 (79.5)
Drug-induced BrS, *n* (%)	9 (20.5)
Family history of SCD, *n* (%)	29 (65.9)
Arrhythmic syncope, *n* (%)	3 (6.8)
Non-arrhythmic syncope, *n* (%)	7 (15.9)
Aborted sudden cardiac death, *n* (%)	2 (4.5)
Nocturnal agonal respiration, *n* (%)	9 (20.5)
Positive genetic test *, *n*/*N* (%)	9/30 (30)
ICD implantation, *n* (%)	17 (38.6)
Primary prevention ICD, *n* (%)	13 (29.5)
Secondary prevention ICD, *n* (%)	4 (9.1)
ICD shock, *n*/*N* (%)	8/17 (47.1)
Appropriate ICD shock, *n*/*N* (%)	3/17 (17.6)
Inappropriate ICD shock, *n*/*N* (%)	6/17 (35.3)
EPS, *n* (%)	25 (56.8)
VRP < 200 ms, *n*/*N* (%)	4/17 (23.5)
Inducible ventricular arrhythmias, *n*/*N* (%)	11/25 (44)

* Genetic test was considered positive when a pathogenic or likely pathogenic variant was identified. BrS = Brugada syndrome; EPS = electrophysiological study; ICD = implantable cardioverter defibrillator; VRP = ventricular refractory period.

**Table 2:** Comparison of clinical characteristics according to BrP1 behavior during tilt table testing.

Variable	Group			*p*
	A	B	C	
Number of patients, *n* (%)	30 (68.1)	5 (11.3)	9 (20.4)	NA
Age, years, mean ± SD	51.1 ± 15.8	52.8 ± 6	40.2 ± 11.4	0.126
Female sex, *n* (%)	11 (36.7)	0 (0)	1 (11.1)	0.054
Family history of SCD, *n* (%)	19 (63.3)	3 (60)	7 (77.8)	0.681
Syncope during tilt test, *n* (%)	5 (16.6)	2 (40)	1 (11.1)	0.380
Syncope type during tilt test				0.191
- Mixed, *n*/*N* (%)	1 (20)	0 (0)	0 (0)	-
- Cardioinhibitory, *n*/*N* (%)	0 (0)	1 (50)	1 (100)	-
- Vasodepressor, *n*/*N* (%)	3 (60)	1 (50)	0 (0)	-
- Exaggerated response to nitrate administration, *n*/*N* (%)	1 (20)	0 (0)	0 (0)	-
Aborted sudden cardiac death, *n* (%)	2 (6.7)	0 (0)	0 (0)	0.455
Nocturnal agonal respiration, *n* (%)	7 (23.3)	0 (0)	2 (22.2)	0.293
Arrhythmic syncope, *n* (%)	0 (0)	1 (20)	2 (22.2)	**0.025**
Non-arrhythmic syncope, *n* (%)	6 (20)	1 (20)	0 (0)	0.171
Positive genetic test *, *n*/*N* (%)	3/17 (17.6)	2/5 (40)	4/4 (50)	0.225
ICD implantation, *n* (%)	8 (26.7)	1 (20)	7 (87.5)	**0.010**
- Primary prevention ICD, *n*/*N* (%)	6 (20)	1 (20)	6 (66.7)	-
- Secondary prevention ICD, *n*/*N* (%)	2 (6.7)	0 (0)	2 (22.2)	-
ICD shock, *n*/*N* (%)	3/8 (37.5)	0/1 (0)	5/8 (62.5)	0.311
Appropriate ICD shock, *n*/*N* (%)	2/8 (25)	0/1 (0)	1/8 (12.5)	0.664
Inappropriate ICD shock, *n*/*N* (%)	2/8 (25)	0/1 (0)	4/8 (50)	0.370
EPS, *n* (%)	18 (60)	2 (40)	6 (66.7)	NA
VRP < 200 ms, *n*/*N* (%)	3/11 (27.3)	0/1 (0)	1/5 (20)	0.721
Inducible ventricular arrhythmias, *n*/*N* (%)	6/17 (35.3)	1/1 (50)	4/6 (66.7)	0.404

Values are presented as mean ± SD or *n* (%). *p* values refer to comparisons across groups A, B, and C using likelihood-ratio tests for categorical variables and analysis of variance for continuous variables. *p* values < 0.05 are shown in bold. NA indicates not applicable for descriptive sample size. * Genetic test was considered positive when a pathogenic or likely pathogenic variant was identified. EPS = electrophysiological study; ICD = implantable cardioverter defibrillator; VRP = ventricular refractory period.

## Data Availability

The raw data supporting the conclusions of this article will be made available by the authors on request.

## Abbreviations

The following abbreviations are used in this manuscript:

BrS	Brugada syndrome
BrP1	Type 1 Brugada pattern
EPS	Electrophysiological study
ICD	Implantable cardioverter defibrillator
ICS	Intercostal space
IPL	Inferior precordial leads
SPL	Superior precordial leads
VRP	Ventricular refractory period
